# Supplementary material for: Learning curve and surgical outcome of robotic assisted colorectal surgery with ERAS program
Source: Sci Rep. 2022 Nov 29;12:20566. doi: 10.1038/s41598-022-24665-w (PMC9709162; doi:10.1038/s41598-022-24665-w)
Supplement: Supplementary file 1 — Supplementary Information 1. [file 41598_2022_24665_MOESM1_ESM.zip › LSC Raw data-Ñ[▒K/095/O_ñw╝╨░O▒Kñσ.pdf]

正式摘要(電子簽章：000000200204543)

請選擇...reporter

臺中榮民總醫院

Operation Note

[redacted]

- 手術日期：2020/09/30
- 手術醫師：VS 林俊余，CR 楊曙榮，R 陳瑋夫，CR 林陳彥
- Anesthesia : GE
- Position : Supine
- Aseptic agents : Soap , Betadine Alcohol , Betadine Aqua

Diagnosis

- Pre-operative diagnosis : T colon ca
- Post-operative diagnosis : 1. Rt side T colon cancer s/p lapascopic RH+ D3 dissection
  cT3N1M0
  2. GBS s/p LC

Surgical Indication

- Removal of T-colon tumor and pathology examination

Operation

- Laparoscopic right colectomy+ D3 dissection

Finding

- 1. a ulcerative tumor at T-colon about 6x7cm in size
  2. some palpable LN at IC and middle colic root
  3. intra coporal anastomosis was done.
  4. penroce drain at subhepatic space
  5. LC was done
  6. Hylobarrer +

Procedure

- Under GE anestheia, the patient was put in supine position. A 1 cm in length skin incision was made at umbilcus and the peritoneal cavity was entered and pneumoperitonium was created. The Endo-Eye Olympus laparoscope were inserted through periumbilicus PORT. another 5mm portx3 and 12 cm port was inserted. The appendix ,cecum and ileocolic vessles were identified one by one. The Ileocolic vessels was dissected, skeletonized and transectioned by clipping and ligasure+ hoook scalpel. The retroperitoneal dissection was done through embryonic plane between meso-right colon and Gerota fascia. The Duodenum and pancrease head was identified and protected. Finally, the right side colon was fully mobilized. Intracoporeal resection of ileum and A-T colon with side to side anastomosis was done by endo GIA 60/3.8mm x5. Penroce drain was inserted and the umbilical wound was closed in layers. Throughout the whole course of operation, little blood loss and patient stood well. The GS consult for LC.
- Estimated blood loss: minimum
- 器械數量: 正確
- 紗布數量: 正確

Image

- PAINT
  - abdomen

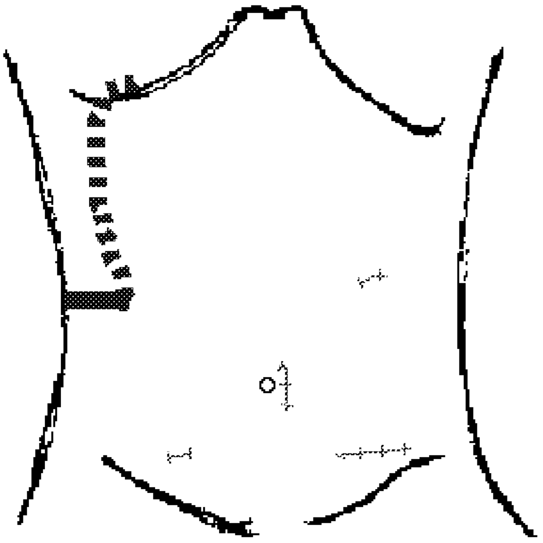

- colon & rectum

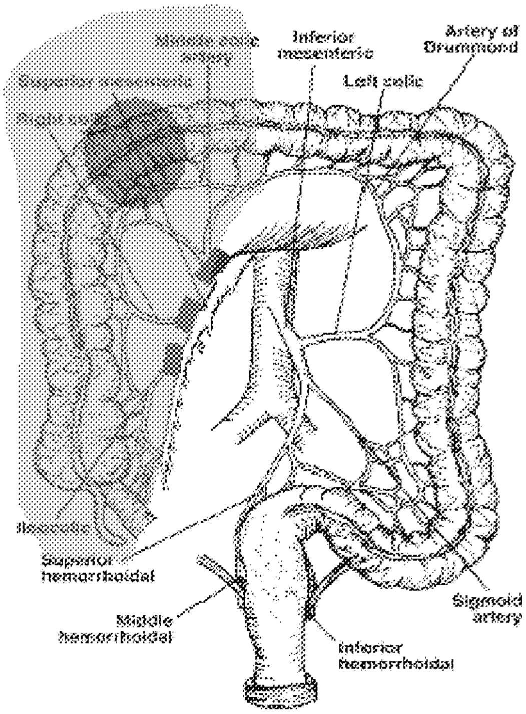

主治 2222H林俊余 / 醫師 2292G楊曙榮

正式摘要(**電子簽章：000000200204543)**

請選擇...reporter

doctorRank:B
